# Caffeine Consumption Helps Honey Bees Fight a Bacterial Pathogen

**DOI:** 10.1128/spectrum.00520-23

**Published:** 2023-05-22

**Authors:** Erick V. S. Motta, Ryan L. W. Arnott, Nancy A. Moran

**Affiliations:** a Department of Integrative Biology, University of Texas at Austin, Austin, Texas, USA; China Agricultural University

**Keywords:** *Apis mellifera*, alkaloids, gut microbiome, pathogens

## Abstract

Caffeine has long been used as a stimulant by humans. Although this secondary metabolite is produced by some plants as a mechanism of defense against herbivores, beneficial or detrimental effects of such consumption are usually associated with dose. The Western honey bee, Apis mellifera, can also be exposed to caffeine when foraging at *Coffea* and *Citrus* plants, and low doses as are found in the nectar of these plants seem to boost memory learning and ameliorate parasite infection in bees. In this study, we investigated the effects of caffeine consumption on the gut microbiota of honey bees and on susceptibility to bacterial infection. We performed *in vivo* experiments in which honey bees, deprived of or colonized with their native microbiota, were exposed to nectar-relevant concentrations of caffeine for a week, then challenged with the bacterial pathogen Serratia marcescens. We found that caffeine consumption did not impact the gut microbiota or survival rates of honey bees. Moreover, microbiota-colonized bees exposed to caffeine were more resistant to infection and exhibited increased survival rates compared to microbiota-colonized or microbiota-deprived bees only exposed to the pathogen. Our findings point to an additional benefit of caffeine consumption in honey bee health by protecting against bacterial infections.

**IMPORTANCE** The consumption of caffeine is a remarkable feature of the human diet. Common drinks, such as coffee and tea, contain caffeine as a stimulant. Interestingly, honey bees also seem to like caffeine. They are usually attracted to the low concentrations of caffeine found in nectar and pollen of *Coffea* plants, and consumption improves learning and memory retention, as well as protects against viruses and fungal parasites. In this study, we expanded these findings by demonstrating that caffeine can improve survival rates of honey bees infected with Serratia marcescens, a bacterial pathogen known to cause sepsis in animals. However, this beneficial effect was only observed when bees were colonized with their native gut microbiota, and caffeine seemed not to directly affect the gut microbiota or survival rates of bees. Our findings suggest a potential synergism between caffeine and gut microbial communities in protection against bacterial pathogens.

## INTRODUCTION

Many bioactive plant-derived metabolites consumed by humans are produced by plants as a mechanism of defense against herbivores (1). Although high doses of these metabolites can be lethal (2, 3), low doses may exhibit pharmacological effects on animal behavior, by enhancing cognitive performance and memory retention (4, 5). Interestingly, some of these metabolites are not only found in leaf and seed tissues, where they could provide a role against herbivory, but are also found at lower doses in nectar and pollen, which are usually rewards for insect pollinators. In this last case, plant metabolites can influence behavior (5) and health (6, 7) of pollinators.

Caffeine (1,3,7-trimethylxanthine) is a bioactive purine alkaloid produced by different plant species, including those used to make coffee (*Coffea*), chocolate, tea, and mate (5, 8–11), and even in *Citrus* trees, suggesting an ecological role in pollinator attraction (5, 12) or herbivore defense (2, 11), depending on plant tissue location and concentration. For example, caffeine has been detected in the nectar and pollen of *Citrus* and *Coffea* species at concentrations up to 1.1 mM (13) and 6.7 mM (9), respectively. These concentrations are much lower than the ones found in leaves and seeds of *Coffea*, which have been reported to be as high as 124 mM (8). Caffeine is bitter tasting to several animals, but bees are not usually deterred by caffeine in nectar, and actually they prefer sucrose solutions containing naturally occurring concentrations of caffeine (14–17). However, when caffeine is present in doses higher than 1 mM, bees can be deterred by the bitter taste (5).

Besides its well-known stimulant effect in humans, caffeine triggers specific behavioral and physiological effects in other animals, including invertebrate pollinators (5, 11, 14). Caffeine consumption improves learning and memory reward formation (5, 18–22) and increases pollination by both honey bees (5, 14, 23, 24) and bumble bees (15), but not by the stingless bee *Plebeia droryana* (25). However, this increased persistence and specificity to a forage location may lead to suboptimal foraging strategies on caffeinated nectar sources with low sugar content (24, 26). Moreover, naturally occurring concentrations of caffeine may help to ameliorate effects caused by insecticide exposure in bees (27–29), and they do not impact survival rates of larvae or worker bees (30), sometimes even improving survival rates (22).

Plant secondary metabolites in nectar and pollen are also associated with parasite tolerance (31–33). Caffeine, for example, can diminish the severity of fungal disease by reducing spore density of the microsporidian *Vairimorpha* spp. (previously known as *Nosema* spp. [34]) and increasing longevity in both honey bees (31, 35) and bumble bees (36), although this is not observed for the trypanosomatid parasite *Crithidia bombi* (37). Low doses of caffeine also impact virus infection (38, 39), by increasing the expression of immunity genes and reducing the levels of Deformed wing virus in honey bees (38) and increasing survival rates of honey bees infected with Israeli acute paralysis virus (39).

The effects of caffeine on bacterial growth have also been tested, with several bacteria being able to grow in the presence of 13 mM caffeine (40). However, bacterial inhibition is observed at higher concentrations, such as 50 mM (41). Interestingly, the gut microbiota of a major insect pest can mediate caffeine detoxification as a mechanism to deal with the high concentrations of caffeine found in *Coffea* plants (42), and there is some evidence that caffeine consumption can initially increase the diversity and abundance of the honey bee gut microbiota, which is followed by a temporal stabilization of the microbiota (43).

In this study, we investigated the effects of naturally relevant concentrations of caffeine on survival rates, gut microbial communities, and bacterial infection susceptibility of the Western honey bee, Apis mellifera. We performed experiments with hive bees and with age-controlled bees, lacking or containing a native microbiota, to investigate how caffeine consumption and the gut microbiota protect bees against an opportunistic bacterial pathogen, Serratia marcescens, known to cause disease and decrease survival rates of honey bees at both larval (44) and adult stages (45, 46).

## RESULTS

**FIG 1:**
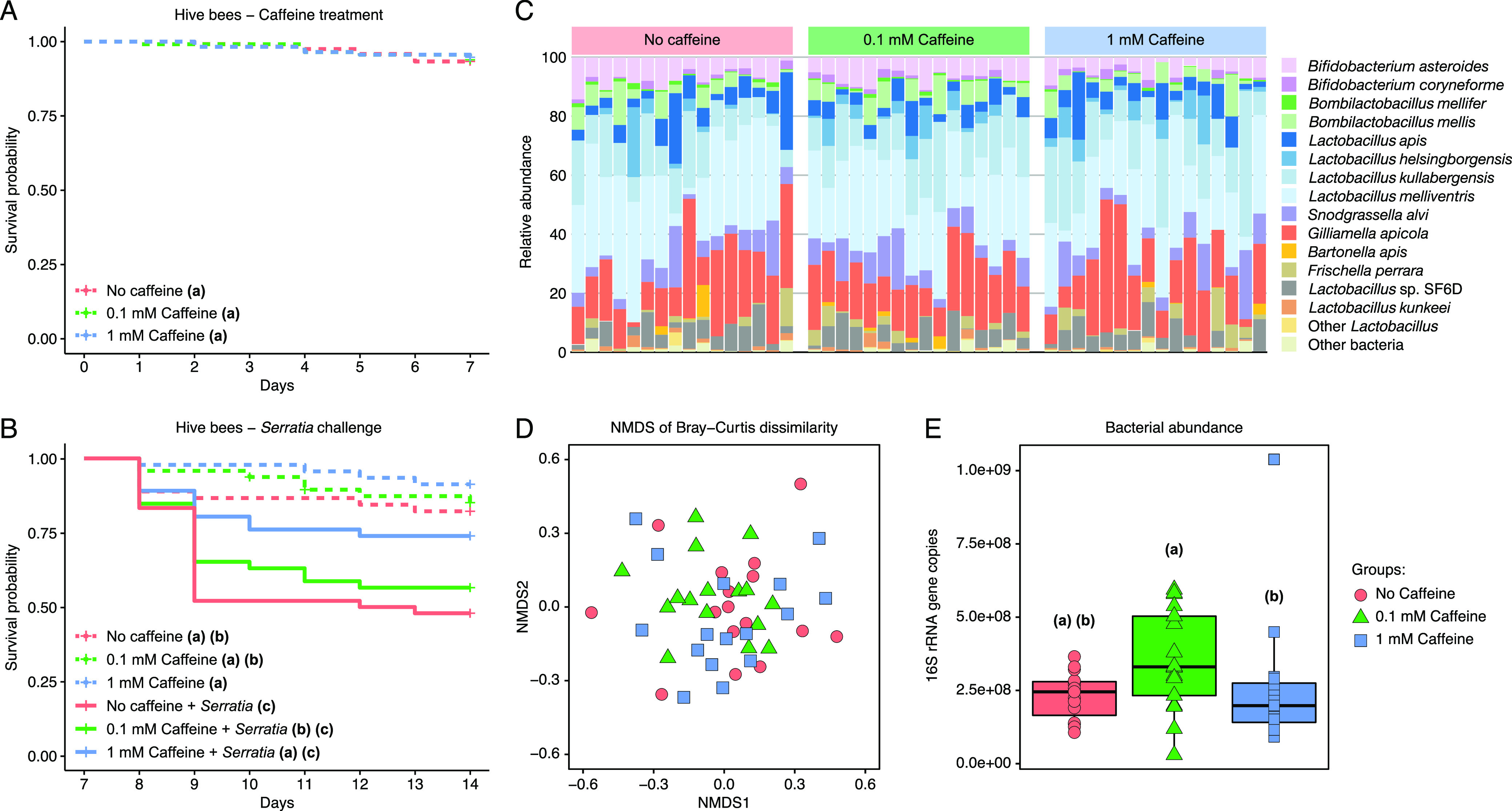
Effects of caffeine consumption on survival rates, pathogen susceptibility, and gut microbial communities of honey bees from the first trial. (A) Kaplan-Meier curves showing the survival probability of hive-collected honey bees (not age-controlled) fed sucrose syrup, 0.1 mM caffeine, or 1 mM caffeine (*n* = 4 cages per group, ~29 bees per cage). (B) Kaplan-Meier curves showing the survival probability of honey bees challenged or not with Serratia marcescens kz19 after treatment with sucrose syrup, 0.1 mM caffeine, or 1 mM caffeine (*n* = 3 cup cages per group, 15 to 16 bees per cup cage). Letters in parentheses on the right side of each group name indicate statistical significance for pairwise comparisons. Groups that do not share a letter are significantly different (ANOVA test, COXME model, *P* < 0.01). (C) Stacked column graph showing the relative abundance of gut bacterial species in honey bee workers fed sucrose syrup, 0.1 mM caffeine, or 1 mM caffeine for 7 days. Each column represents one bee (*n* = 16 bees per group). (D) Nonmetric multidimensional scaling of Bray-Curtis dissimilarity of gut community compositions of honey bees fed sucrose syrup, 0.1 mM caffeine, or 1 mM caffeine for 7 days (*n* = 16 bees per group). (E) Boxplots of total bacterial 16S rRNA gene copies in the guts of bees fed sucrose syrup, 0.1 mM caffeine, or 1 mM caffeine for 7 days (*n* = 16 bees per group). Letters in parentheses above each boxplot indicate statistical significance for pairwise comparisons. Groups that do not share a letter are significantly different (Kruskal-Wallis test followed by Dunn’s multiple-comparison test, *P* < 0.05).

To investigate the effects of caffeine consumption on the gut microbiota and on the survival rates of honey bees, we performed three trials of a two-step *in vivo* experiment in which bees were first treated with caffeine and then challenged with S. marcescens. In the first trial, we used bees of various ages collected from inside a hive. During the caffeine treatment step, we did not observe changes in survival rates between control and caffeine-treated bees after 7 days (analysis of variance [ANOVA] test, COXME model; χ^2^ = 8.61, df = 2, *P* = 0.91, *N* = 348), and survival rates were higher than 90% for all groups (Fig. 1A). However, we observed significant changes in survival rates between groups during the *Serratia* challenge step (ANOVA test, COXME model; χ^2^ = 30.25, df = 5, *P* = 1.32e–05, *N* = 279). Control and caffeine-treated bees not challenged with *Serratia* exhibited higher survival rates than bees challenged with *Serratia* after 7 days (Fig. 1B, Table 1). Interestingly, 1 mM caffeine-treated bees challenged with *Serratia* exhibited nonsignificantly higher survival rates (~74%) than 0.1 mM caffeine-treated bees (~57%) or control bees (~48%) challenged with *Serratia* (Fig. 1B, Table 1). Also, control and caffeine-treated bees not challenged with *Serratia* did not exhibit significant changes in survival rates compared to 1 mM caffeine-treated bees challenged with *Serratia* (Fig. 1B, Table 1), suggesting a potential benefit of 1 mM caffeine consumption in protection against this opportunistic bacterium.

**TABLE 1:** Statistical reports for the first trial with hive bees

Treatment and statistical test	Comparison group	Chi-squared	df	Estimate	SE	*Z* ratio	*N*	*P* value
Caffeine treatment								
ANOVA		0.19	2				348	0.91
Emmeans (pairwise comparisons)								
No caffeine	0.1 mM caffeine			0.08	0.52	0.15		0.99
No caffeine	1 mM caffeine			0.24	0.54	0.44		0.90
0.1 mM caffeine	1 mM caffeine			0.16	0.56	0.29		0.95
*Serratia* challenge								
ANOVA		30.25	5				279	1.32e–05
Emmeans (pairwise comparisons)								
No caffeine	0.1 mM caffeine			−0.01	0.49	−0.02		1.00
No caffeine	1 mM caffeine			0.81	0.61	1.34		0.76
No caffeine	*Serratia*			−1.37	0.40	−3.42		0.01
No caffeine	0.1 mM caffeine + *Serratia*			−1.09	0.41	−2.65		0.08
No caffeine	1 mM caffeine + *Serratia*			−0.45	0.45	−1.00		0.92
0.1 mM caffeine	1 mM caffeine			0.82	0.62	1.33		0.77
0.1 mM caffeine	No caffeine + *Serratia*			−1.36	0.42	−3.26		0.01
0.1 mM caffeine	0.1 mM caffeine + *Serratia*			−1.09	0.43	−2.53		0.12
0.1 mM caffeine	1 mM caffeine + *Serratia*			−0.44	0.47	−0.95		0.93
1 mM caffeine	No caffeine + *Serratia*			−2.18	0.55	−3.99		0.001
1 mM *C*affeine	0.1 mM caffeine + *Serratia*			−1.91	0.56	−3.43		0.01
1 mM *C*affeine	1 mM caffeine + *Serratia*			−1.26	0.58	−2.16		0.26
No caffeine + *Serratia*	0.1 mM caffeine + *Serratia*			0.27	0.31	0.87		0.95
No caffeine + *Serratia*	1 mM caffeine + *Serratia*			0.92	0.36	2.52		0.12
0.1 mM caffeine + *Serratia*	1 mM caffeine + *Serratia*			0.64	0.38	1.70		0.53

In the first trial, we also checked changes in microbial abundance and composition between control and caffeine-treated bees after 7 days of treatment. Processing of 16S rRNA amplicon sequences from bee guts identified the following dominant bacteria in our samples: *Bifidobacterium* spp., *Bombilactobacillus* spp., *Lactobacillus* spp., Snodgrassella alvi, Gilliamella apicola, Bartonella apis, and Frischella perrara (Fig. 1C), which was consistent with previous data from the honey bee gut microbiota (47–49). We found no changes in microbial diversity, based on Bray-Curtis dissimilarity analysis (Fig. 1D) (Adonis test; df = 2, F model = 0.79, *R*^2^ = 0.03, *P* > 0.05). However, we observed changes in total bacterial abundance between groups (Kruskal-Wallis test; χ^2^ = 7.34, df = 2, *P* = 0.03), with bacterial abundance in the guts of 0.1 mM caffeine-treated bees significantly higher than in 1 mM caffeine-treated bees (Fig. 1E) (Dunn’s multiple-comparison test; *Z* = 2.51, *P* = 0.04).

**FIG 2:**
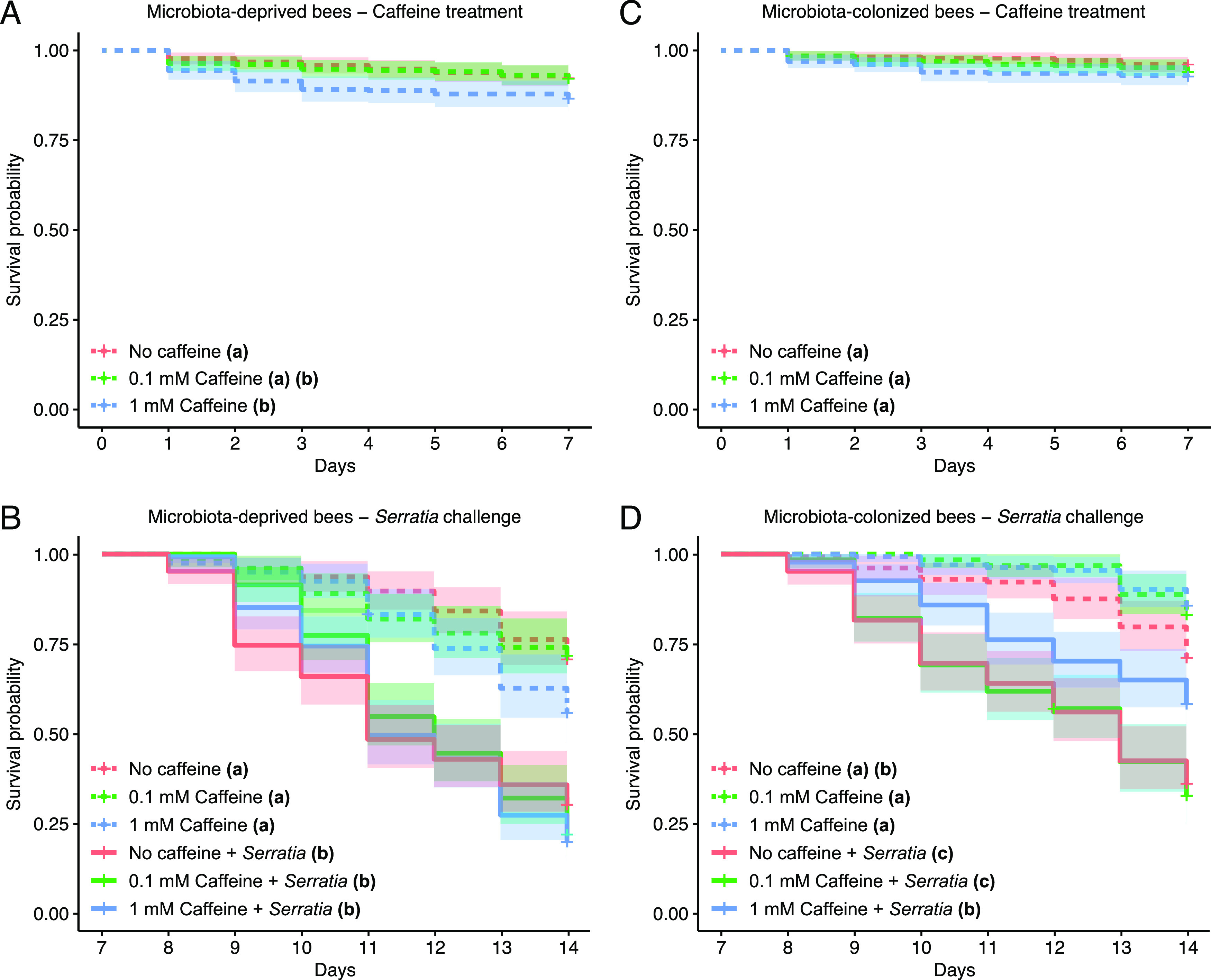
Effects of caffeine consumption and pathogen susceptibility in age-controlled honey bees from the second and third trials. Kaplan-Meier survival curves show the survival probability of microbiota-deprived bees fed sucrose syrup, 0.1 mM caffeine, or 1 mM caffeine (*n* = 2 colonies, 18 cages per colony, 12 cages per group, ~25 bees per cage) (A), of microbiota-deprived bees challenged with Serratia marcescens kz19 after caffeine treatment (*n* = 2 colonies, 18 cages per colony, 6 cages per group, ~21 bees per cage) (B), of microbiota-colonized bees fed sucrose syrup, 0.1 mM caffeine, or 1 mM caffeine (*n* = 2 colonies, 18 cages per colony, 12 cages per group, ~28 bees per cage) (C), and of microbiota-colonized bees challenged with S. marcescens kz19 after caffeine treatment (*n* = 2 colonies, 18 cages per colony, 6 cages per group, ~24 bees per cage) (D). Shaded regions represent 95% confidence intervals for each curve. Letters in parentheses on the right side of each group name indicate statistical significance for pairwise comparisons. Groups that do not share a letter are significantly different (Emmeans, multiple pairwise comparisons, *P* < 0.001).

To further investigate the potential contribution of caffeine treatment in protection against *Serratia*, we performed two additional trials with age-controlled, newly emerged bees, in order to minimize the effects of variable age in our results (Fig. 2). In these replicate trials, we included two major groups of microbiota-deprived and microbiota-colonized bees, to account for and understand the effects of the microbiota during the caffeine treatment and the *Serratia* challenge. In these trials, the caffeine treatment step affected survival rates in microbiota-deprived groups (ANOVA test, COXME model; χ^2^ = 7.76, df = 2, *P* = 0.02, *N* = 919) (Fig. 2A), but not in microbiota-colonized groups (ANOVA test, COXME model; χ^2^ = 3.69, df = 2, *P* = 0.16, *N* = 990) (Fig. 2C). More specifically, microbiota-deprived bees treated with 1 mM caffeine exhibited lower survival rates than control bees (Fig. 2A, Table 2). However, during the *Serratia* challenge step, we observed significant treatment effect on survival rates for both microbiota-deprived (ANOVA test, COXME model; χ^2^ = 136.90, df = 5, *P* = 2.20e–16, *N *= 747) (Fig. 2B) and microbiota-colonized groups (ANOVA test, COXME model; χ^2^ = 142.33, df = 5, *P* = 2.20e–16, *N* = 766) (Fig. 2D). In the microbiota-deprived group, *Serratia* challenge, but not caffeine treatment, significantly impacted survival rates, with pathogen-challenged bees showing lower survival, as expected (Fig. 2B, Table 2). In the microbiota-colonized group, on the other hand, both caffeine treatment and *Serratia* challenge affected survival rates. Bees treated or not with caffeine and not exposed to *Serratia* exhibited similar survival rates to one another and higher survival than control bees or bees treated with 0.1 mM caffeine and exposed to *Serratia* (Fig. 2D, Table 3). Interestingly, 1 mM caffeine-treated bees exposed to *Serratia* exhibited similar survival rates as control bees not exposed to *Serratia* and significantly higher survival rates than control bees or 0.1 mM caffeine-treated bees exposed to *Serratia* (Fig. 2D, Table 3). Although not statistically significant, there was a trend in increased survival rates for microbiota-colonized bees treated with caffeine compared to control bees when the pathogen was absent (Fig. 2D, Table 3).

**TABLE 2:** Statistical reports for the second and third trials with age-controlled, microbiota-deprived bees

Treatment and statistical test	Comparison group	Chi-squared	df	Estimate	SE	*Z* ratio	*N*	*P* value
Caffeine treatment								
ANOVA		7.78	2				919	0.02
Emmeans (pairwise comparisons)								
No caffeine	0.1 mM caffeine			−0.02	0.29	−0.07		1.00
No caffeine	1 mM caffeine			−0.60	0.26	−2.34		0.05
0.1 mM caffeine	1 mM caffeine			−0.58	0.26	−2.27		0.06
*Serratia* challenge								
ANOVA		136.90	5				747	2.20e–16
Emmeans (pairwise comparisons)								
No caffeine	0.1 mM caffeine			−0.05	0.23	−0.21		1.00
No caffeine	1 mM caffeine			−0.50	0.22	−2.32		0.18
No caffeine	No caffeine + *Serratia*			−1.37	0.20	−6.98		<0.0001
No caffeine	0.1 mM caffeine + *Serratia*			−1.44	0.19	−7.43		<0.0001
No caffeine	1 mM caffeine + *Serratia*			−1.55	0.20	−7.95		<0.0001
0.1 mM caffeine	1 mM caffeine			−0.45	0.22	−2.07		0.30
0.1 mM caffeine	No caffeine + *Serratia*			−1.32	0.20	−6.63		<0.0001
0.1 mM caffeine	0.1 mM caffeine + *Serratia*			−1.39	0.20	−7.10		<0.0001
0.1 mM caffeine	1 mM caffeine + *Serratia*			−1.50	0.20	−7.63		<0.0001
1 mM caffeine	No caffeine + *Serratia*			−0.87	0.18	−4.97		<0.0001
1 mM caffeine	0.1 mM caffeine + *Serratia*			−0.94	0.17	−5.46		<0.0001
1 mM caffeine	1 mM caffeine + *Serratia*			−1.05	0.17	−6.06		<0.0001
No caffeine + *Serratia*	0.1 mM caffeine + *Serratia*			0.07	0.15	0.44		1.00
No caffeine + *Serratia*	1 mM caffeine + *Serratia*			0.18	0.15	1.20		0.84
0.1 mM caffeine + *Serratia*	1 mM caffeine + *Serratia*			−0.11	0.14	−0.80		0.97

**TABLE 3:** Statistical reports for the second and third trials with age-controlled, microbiota-colonized bees

Treatment and statistical test	Comparison group	Chi-squared	df	Estimate	SE	*Z* ratio	*N*	*P* value
Caffeine treatment								
ANOVA		3.69	2				990	0.16
*Serratia* challenge								
ANOVA		142.33	5				766	2.20e–16
Emmeans (pairwise comparisons)								
No caffeine	0.1 mM caffeine			0.65	0.29	2.25		0.22
No caffeine	1 mM caffeine			0.84	0.30	2.82		0.06
No caffeine	No caffeine + *Serratia*			−1.38	0.22	−6.20		<0.0001
No caffeine	0.1 mM caffeine + *Serratia*			−1.40	0.22	−6.25		<0.0001
No caffeine	1 mM caffeine + *Serratia*			−0.56	0.23	−2.42		0.15
0.1 mM caffeine	1 mM caffeine			0.19	0.33	0.57		0.99
0.1 mM caffeine	No caffeine + *Serratia*			−2.03	0.27	−7.66		<0.0001
0.1 mM caffeine	0.1 mM caffeine + *Serratia*			−2.05	0.27	−7.69		<0.0001
0.1 mM caffeine	1 mM caffeine + *Serratia*			−1.21	0.27	−4.44		0.0001
1 mM caffeine	No caffeine + *Serratia*			−2.21	0.27	−8.08		<0.0001
1 mM caffeine	0.1 mM caffeine + *Serratia*			−2.24	0.28	−8.12		<0.0001
1 mM caffeine	1 mM caffeine + *Serratia*			−1.40	0.28	−4.97		<0.0001
No caffeine + *Serratia*	0.1 mM caffeine + *Serratia*			0.03	0.19	0.14		1.00
No caffeine + *Serratia*	1 mM caffeine + *Serratia*			−0.82	0.20	−4.08		0.0006
0.1 mM caffeine + *Serratia*	1 mM caffeine + *Serratia*			0.84	0.20	4.16		0.0005

## DISCUSSION

Plant-derived metabolites have long been used by humans as prophylactic or therapeutic medication for the treatments of diseases (50, 51). This is also true for other animals, including insect pollinators (52, 53). Bees, for example, collect metabolites from plant resins, nectar, and pollen as a self-medication mechanism against parasites (6, 54). In our study, we were particularly interested in investigating the effects of caffeine on honey bee susceptibility to bacterial infection. We found that caffeine consumption helped honey bees to better survive infection by the opportunistic bacteria S. marcescens. This finding contributes to the understanding of the beneficial effects of low doses of caffeine in animal health and protection against disease. Caffeine, as a neuroactive alkaloid, can affect both the behavior and physiology of bees (5), but it can also provide a beneficial role in protection against parasites, such as *Vairimorpha* spp. (31, 35, 36), and viruses, such as Deformed wing virus and Israeli acute paralysis virus (38, 39), increasing the survival rates of infected bees.

S. marcescens is an opportunistic bacterium of honey bees that has been reported to cause diseases at both larval (44) and adult stages (45, 46). This bacterium also increases mortality rates of adult bees exposed to antibiotics (55). Our findings, showing that caffeine consumption improves survival rates of honey bees following exposure to S. marcescens, raise the question of whether caffeine can also provide a similar benefit to other bees or honey bees exposed to other bacterial pathogens, such as Bacillus pulvifaciens, which causes powdery scale, and *Spiroplasma* spp., involved in May disease (56, 57). Moreover, the most discussed bacterial infections in honey bees are associated with brood diseases, primarily involving American and European foulbrood, which are caused by Paenibacillus larvae and Melissococcus plutonius, respectively (58, 59), and future studies should continue investigating the roles of specific plant-derived metabolites, such as caffeine, in the treatment of bacterial infections affecting brood development (60).

As important as the finding of protection against pathogens is the finding that exposure to low doses of caffeine has no evident fitness cost to bees. Survival rates of caffeine-exposed bees usually increase (22) or remain the same (30), at least under concentrations found in nectar. In our study, caffeine exposure did not affect survival rates of honey bees, although we observed a trend toward increased survival rates. However, caffeine, similar to other alkaloids, has dose-dependent effects on pollinator behavior and activity, and exposure to concentrations similar to what is found in leaves and seeds can be toxic and negatively impact animal health (61). Since bees collect plant resins to make propolis (54), concentrations of caffeine in these resources should be also taken into consideration. Indeed, high concentrations of caffeine may act as a deterrent and may even be lethal to bees (62). In other animals, such as caterpillars, parasitized individuals prefer diets rich in toxic plant metabolites, which improve their survival rates, though this reduces fitness of uninfected individuals (63, 64).

We investigated the effects of caffeine on gut microbial communities of honey bees and did not find an impact on the abundance or composition of the main bacterial species in the bee gut, suggesting that caffeine has little or no impact on the honey bee gut microbiota. This finding is important because the gut microbiota plays important roles in bee health, contributing to immune system regulation (48, 65, 66), detoxification (67, 68), digestion of recalcitrant components of pollen (69, 70), and protection against pathogens (71–73). Furthermore, perturbations of the gut microbiota can lead to immune system dysregulation (74, 75) and increased susceptibility to infection (55, 74, 76). However, a previous study found an impact of caffeine consumption on the diversity and abundance of the honey bee gut microbiota, but this impact only occurred during the first few days of exposure (43). Since we only checked potential microbiota changes after a week of exposure and only used 16S rRNA amplicon sequencing, we may have missed a potential effect in the beginning of treatment or on the fine-scale strain diversity of the honey bee microbiota.

We also performed experiments with microbiota-deprived bees and microbiota-colonized bees, and the protective effect of caffeine against S. marcescens was only observed in microbiota-colonized bees. These results demonstrate that an intact microbiota plays a crucial role in protection against pathogens (71, 73), as has been observed in cases of dysbiosis in honey bees (55, 74, 77). The molecular mechanisms behind the synergistic effect of caffeine and the gut microbiota in protection against S. marcescens deserves further investigation. They may reflect interactions between caffeine and specific members of the gut microbiota. In some organisms, such as the berry borer (or coffee borer beetle), caffeine is metabolized by the microbiota, which allows this pest to feed on and damage coffee beans (42). Further studies are needed to investigate whether members of the bee gut microbiota metabolize caffeine and whether this underlies the beneficial effects described here and in previous studies. Moreover, direct effects of caffeine on S. marcescens cannot be dismissed, as previous studies found that high concentrations of caffeine inhibited this opportunistic pathogen *in vitro* (78). It is also known that different bacterial species can metabolize caffeine (79), including some strains of S. marcescens (80).

### Conclusions.

Our findings demonstrate that caffeine consumption does not affect gut microbial communities or survival rates of honey bees. Interestingly, specific concentrations of caffeine can improve survival rates of microbiota-colonized honey bees exposed to the bacterial pathogen S. marcescens compared to bees not exposed to caffeine. Since this effect is not observed for microbiota-deprived bees, the establishment of the native gut microbiota must be crucial for caffeine to exert a protective effect against *Serratia* in bees. These findings expand previously established evidence that caffeine ingestion increases honey bee protection against natural enemies, such as fungal parasites and viruses, as our findings showed protection against an opportunistic bacterial pathogen.

## MATERIALS AND METHODS

### Honey bee rearing.

Honey bees (*A. mellifera*) were obtained from outdoor hives kept on the rooftop of J. T. Patterson Laboratories Building at the University of Texas at Austin (latitude, 30.287913; longitude, 97.736183). These hives are self-sufficient.

### Treatment preparation.

Caffeine standard (99.7% purity, lot number W24A011) was purchased from Alfa Aesar by Thermo Fisher Scientific. Stock solutions of 10 mM caffeine were prepared with heated molecular biology-grade water and used to prepare working solutions with final concentrations of 0.1 mM or 1 mM caffeine in filter-sterilized 0.5 M sucrose syrup.

### Caffeine exposure and *Serratia* challenge experiments.

**(i) First trial.** Approximately 360 honey bee workers (not age controlled) were collected from inside a hive in fall 2017. They were brought into the lab, immobilized at 4°C, and randomly transferred to single-use cages constructed from plastic cups (81). Bees were in groups of 28 to 30 bees in each of 12 cup cages. Cup cages were split into three treatment groups, which were provided (i) sterile sucrose syrup, (ii) 0.1 mM caffeine in sterile sucrose syrup, or (iii) 1 mM caffeine in sterile sucrose syrup. Survival rates were monitored for 7 days, and dead bees were removed in a daily census. After that, treatments were removed, and 16 bees were sampled from each treatment group and preserved at −80°C. The remaining bees were briefly immobilized with carbon dioxide, mixed according to treatment group, and then transferred to new cup cages in groups of 15 to 16 bees for a total of six cup cages per treatment group. Finally, each treatment group was divided into two subgroups: one subgroup was used as a control and fed sterile sucrose syrup, whereas the other group was challenged with the opportunistic pathogen Serratia marcescens strain kz19 in sucrose syrup. Survival rates were monitored for 7 days, and dead bees were removed in a daily census.

**(ii) Second trial.** Thousands of late-stage pupae, with pigmented eyes but lacking movement, were removed from brood frames from a different hive in fall of 2020. Pupae were transferred to clean plastic bins and placed in an incubator at 35°C and ~60% relative humidity to simulate hive conditions until emerging as adults. Approximately 1,200 healthy newly emerged workers (age controlled) were randomly transferred to cup cages in groups of 30 to 35 bees for a total of 36 cup cages. These cup cages were first split into two major groups. Bees from one major group were allowed to acquire their normal microbiota by addition of a suspension of freshly prepared gut homogenate (see below) from hive bees to sterile pollen, while bees from the other major group were prevented from acquiring microbiota and provided only sterile pollen. Then, cup cages from each major group were divided into three treatment groups and fed sterile sucrose syrup, 0.1 mM caffeine in sterile sucrose syrup, or 1 mM caffeine in sterile sucrose syrup. Survival rates were monitored for 7 days, and dead bees were removed in a daily census. Finally, each treatment group was divided into two subgroups. One subgroup was used as a control and provided only sterile sucrose syrup, whereas the other subgroup was challenged with the opportunistic pathogen S. marcescens kz19 in sucrose syrup. Survival rates were monitored for an additional 7 days, and dead bees were removed in a daily census.

**(iii) Third trial.** Thousands of late-stage pupae were removed from brood frames from a different hive in fall of 2022 and managed similar to the second trial. Approximately 960 healthy newly emerged workers were randomly transferred to cup cages in groups of 20 bees for a total of 48 cup cages. Cup cages were split into two major groups, microbiota-deprived and microbiota-colonized groups, then split into three treatment groups which were fed sterile sucrose syrup, 0.1 mM caffeine in sterile sucrose syrup, or 1 mM caffeine in sterile sucrose syrup. Survival rates were monitored for 7 days, and dead bees were removed in a daily census. Cup cages in which we observed high mortality rates due to syrup leakage or feeding tube clogging were removed from the following step and analyses. Then, cup cages from each treatment group were divided into two subgroups to be used as controls or challenged with S. marcescens kz19. Survival rates were monitored for an additional 7 days, and dead bees were removed in a daily census.

### Preparation of gut homogenates.

For the second and third trials, half of the bees were allowed to acquire a normal microbiota by providing gut homogenate solutions mixed with irradiated pollen. Gut homogenate solutions were prepared by aseptically pulling out guts from healthy workers from the same hive and homogenizing them with equal proportions of 1× phosphate-buffered saline (PBS) and sterile sucrose syrup. For each cup from the microbiota-colonized group, 200 μL of gut homogenate solution was mixed with irradiated pollen and provided in small troughs. For each cup from the microbiota-deprived group, only 200 μL of 1:1 PBS-sterile sucrose syrup solution was mixed with irradiated pollen.

### Preparation of Serratia marcescens suspension.

For each trial, a S. marcescens kz19 suspension in sucrose syrup with an optical density (OD) of 0.5 was prepared and provided in feeding tubes to half of the cup cages in each treatment group. S. marcescens kz19 was grown in LB broth at 37°C overnight. Then, the OD was measured at 600 nm, and bacterial cells were diluted to an OD of 0.5 in 1:10 PBS-sucrose syrup and provided to the cup cages from the *Serratia* challenge group. For consistency, cup cages from the control group were provided 1:10 PBS-sucrose syrup.

### DNA extraction.

Honey bees sampled from the first trial (16 bees from each group, 48 bees in total) were dissected with sterilized forceps under aseptic conditions, and DNA was extracted from individual bee guts using a previously described protocol (82), with some adaptations. Briefly, guts were homogenized with 100 μL of cetyl triethylammonium bromide (CTAB) buffer (0.1 M Tris-HCl [pH 8.0], 1.4 M NaCl, 20 mM EDTA, and 20 mg/mL cetrimonium bromide), resuspended in additional 600 μL of CTAB buffer and 20 μL of proteinase K solution (0.1 M Tris-HCl, 26 mM CaCl_2_, 50% glycerol, and 20 mg/mL proteinase K), and transferred to a capped vial with 0.5 mL of 0.1 mm zirconia beads (BioSpec Products Inc.). After adding 2 μL of 2-mercaptoethanol and 2 μL of RNase A cocktail (Invitrogen Corp.), samples were bead-beated for 2 × 2 min. Samples were digested overnight at 50°C and then mixed with 750 μL of phenol-chloroform-isoamyl alcohol (25:24:1, pH 8.0). Samples were inverted five times and centrifuged for 15 min at 4°C and 14,000 rpm. The aqueous layer was transferred to a new vial, and DNA was precipitated at −20°C for 30 min with 700 μL of isopropanol and 70 μL of 3 M NaOAc (pH 5.4). Precipitated samples were centrifuged for 30 min at 4°C and 14,000 rpm, and the supernatant was removed. DNA pellets were washed with 1 mL of cold 75% ethanol and centrifuged for additional 3 min at 4°C. After removing the ethanol wash, and the DNA pellets were dried at room temperature for ~30 min and then resuspended in 50 μL of water. Final DNA samples were stored at −20°C.

### qPCR analysis.

DNA samples from the first trial (48 in total) were 100-fold diluted to be used as the templates for quantitative PCR (qPCR) analysis. For these measures, 10-μL triplicate reaction mixtures were carried out on 96-well plates on an Eppendorf Mastercycler ep realplex instrument using 5 μL of iTaq Universal SYBR green Supermix (Bio-Rad Inc.), 0.05 μL of 100 μM forward and reverse primers (27F, 5′-AGAGTTTGATCCTGGCTCAG-3′; 355R, 5′-CTGCTGCCTCCCGTAGGAGT-3′), 3.9 μL of H_2_O, and 1.0 μL of diluted DNA. The cycling conditions consisted of an initial step of 95°C for 3 min, followed by 5 cycles of a three-step PCR (95°C for 5 s, 65 to 60°C for 15 s with decrease of 1°C per cycle, and 68°C for 20 s), then 35 cycles of a second three-step PCR (95°C for 5 s, 60°C for 15 s, and 68°C for 20 s). Total bacterial 16S rRNA gene copies were estimated by standard curves from amplification of the cloned target sequence in a pGEM-T vector (Promega), as described elsewhere (83).

### 16S rRNA amplicon sequencing.

DNA samples from the first trial (48 in total) were used to investigate bacterial community profiling based on sequencing of the V4 region of the 16S rRNA gene, as amplified by PCR primers Hyb515F (5′-TCGTCGGCAGCGTCAGATGTGTATAAGAGACAGGTGYCAGCMGCCGCGGTA-3′) and Hyb806R (5′-GTCTCGTGGGCTCGGAGATGTGTATAAGAGACAGGGACTACHVGGGTWTCTAAT-3′). Amplification, library preparation, and sequencing (Illumina MiSeq 2 × 250) were performed by the Genomic Sequencing and Analysis Facility at the University of Texas at Austin.

### Bioinformatic analyses.

Illumina sequence reads were processed in QIIME 2 version 2019.10 (84). Primer and adapter sequences were removed using the cutadapt plugin (85). Forward and reverse reads were joined and filtered to remove low-quality reads. Joined reads were truncated to a length of 230 bp and denoised, and chimeric reads were removed using the Deblur plugin (86). Taxonomy was assigned to amplicon sequence variants (ASVs) using the SILVA database in the feature-classifier plugin (87). When necessary, BLASTn searches were conducted against the NCBI database (November 2022). Reads with <0.1% abundance were removed using the feature-table plugin, as were unassigned, mitochondrial, and chloroplast reads using the taxa filter-table plugin. A table of ASVs was generated to investigate changes in microbial abundance and composition between control and treatments (see the supplemental material).

### Statistical analyses.

Comparisons of survival rates between control and treatment groups during caffeine exposure and *Serratia* challenge were performed using mixed-effects Cox proportional hazards models, which were fitted using the function coxme in the R package coxme (88). For the first trial, treatment was considered a fixed effect, and bees within cup cages were considered random effects. The following formula was used: survival (start, stop, death) ~ treatment + [1|(cage/bee)]. For the second and third trials, which were replicates and analyzed together, treatment was considered a fixed effect and bees within cup cages within colonies as random effects. The following formula was used: survival (start, stop, death) ~ treatment + [1|(colony/cage/bee)]. Survival curves were estimated and plotted using the Kaplan-Meier method and the functions survfit and ggsurvplot implemented in the R package survminer (89). Statistical analyses were performed for the fitted models using the function ANOVA in the R package car (90). If significant, multiple pairwise comparisons were performed using the R package emmeans (91), which uses the Tukey method for *P* value adjustment.

Microbial diversity analysis was performed to investigate the effect of caffeine exposure on the microbiota of sampled honey bees. Nonmetric multidimensional scaling based on Bray-Curtis dissimilarity was plotted using the R package phyloseq (92). Statistical tests were performed using the Adonis method for permutational multivariate analysis of variance in the R package vegan (93).

Comparisons of changes in the abundance of gut bacteria between control and treatment groups were performed using the nonparametric Kruskal-Wallis test followed by Dunn’s multiple-comparison test, if significant, in R version 3.5.2 (94).

### Data availability.

Sequencing data are available at NCBI BioProject PRJNA905699. Other data are included in the published article and its supplemental material file.

## References

[1] Sullivan RJ, Hagen EH, Hammerstein P. 2008. Revealing the paradox of drug reward in human evolution. Proc Biol Sci 275:1231–1241. doi:10.1098/rspb.2007.1673. 18353749 PMC2367444

[2] Hollingsworth RG, Armstrong JW, Campbell E. 2002. Caffeine as a repellent for slugs and snails. Nature 417:915–916. doi:10.1038/417915a. 12087394

[3] Rudolph T, Knudsen K. 2010. A case of fatal caffeine poisoning. Acta Anaesthesiol Scand 54:521–523. doi:10.1111/j.1399-6576.2009.02201.x. 20096021

[4] Nehlig A. 1999. Are we dependent upon coffee and caffeine? A review on human and animal data. Neurosci Biobehav Rev 23:563–576. doi:10.1016/s0149-7634(98)00050-5. 10073894

[5] Wright GA, Baker DD, Palmer MJ, Stabler D, Mustard JA, Power EF, Borland AM, Stevenson PC. 2013. Caffeine in floral nectar enhances a pollinator’s memory of reward. Science 339:1202–1204. doi:10.1126/science.1228806. 23471406 PMC4521368

[6] Erler S, Moritz RFA. 2016. Pharmacophagy and pharmacophory: mechanisms of self-medication and disease prevention in the honeybee colony (Apis mellifera). Apidologie 47:389–411. doi:10.1007/s13592-015-0400-z.

[7] Fitch G, Figueroa LL, Koch H, Stevenson PC, Adler LS. 2022. Understanding effects of floral products on bee parasites: mechanisms, synergism, and ecological complexity. Int J Parasitol Parasites Wildl 17:244–256. doi:10.1016/j.ijppaw.2022.02.011. 35299588 PMC8920997

[8] Ashihara H, Crozier A. 2001. Caffeine: a well known but little mentioned compound in plant science. Trends Plant Sci 6:407–413. doi:10.1016/s1360-1385(01)02055-6. 11544129

[9] Kretschmar JA, Baumann TW. 1999. Caffeine in Citrus flowers. Phytochemistry 52:19–23. doi:10.1016/S0031-9422(99)00119-3.

[10] Naef R, Jaquier A, Velluz A, Bachofen B. 2005. From the linden flower to linden honey: volatile constituents of linden nectar, the extract of bee-stomach and ripe honey, p 31–40. *In* Kraft P, Swift KAD (ed), Perspectives in flavor and fragrance research. John Wiley & Sons, Hoboken, NJ. 10.1002/cbdv.200490143 17191825

[11] Nathanson JA. 1984. Caffeine and related methylxanthines: possible naturally occurring pesticides. Science 226:184–187. doi:10.1126/science.6207592. 6207592

[12] Gross M. 2021. What coffee does to body and mind. Curr Biol 31:R311–R313. doi:10.1016/j.cub.2021.03.080.

[13] Prado SG, Collazo JA, Stevenson PC, Irwin RE. 2019. A comparison of coffee floral traits under two different agricultural practices. Sci Rep 9:7331. doi:10.1038/s41598-019-43753-y. 31089179 PMC6517588

[14] Singaravelan N, Nee'man G, Inbar M, Izhaki I. 2005. Feeding responses of free-flying honeybees to secondary compounds mimicking floral nectars. J Chem Ecol 31:2791–2804. doi:10.1007/s10886-005-8394-z. 16365705

[15] Thomson JD, Draguleasa MA, Tan MG. 2015. Flowers with caffeinated nectar receive more pollination. Arthropod Plant Interact 9:1–7. doi:10.1007/s11829-014-9350-z.

[16] Tiedeken EJ, Stout JC, Stevenson PC, Wright GA. 2014. Bumblebees are not deterred by ecologically relevant concentrations of nectar toxins. J Exp Biol 217:1620–1625. 24526720 10.1242/jeb.097543 PMC4006588

[17] Arnold SEJ, Dudenhöffer J-H, Fountain MT, James KL, Hall DR, Farman DI, Wäckers FL, Stevenson PC. 2021. Bumble bees show an induced preference for flowers when primed with caffeinated nectar and a target floral odor. Curr Biol 31:4127–4131.e4. doi:10.1016/j.cub.2021.06.068. 34324835

[18] Gong Z, Gu G, Wang Y, Dong S, Tan K, Nieh JC. 2021. Floral tea polyphenols can improve honey bee memory retention and olfactory sensitivity. J Insect Physiol 128:104177. doi:10.1016/j.jinsphys.2020.104177. 33279470

[19] Mustard JA. 2014. The buzz on caffeine in invertebrates: effects on behavior and molecular mechanisms. Cell Mol Life Sci 71:1375–1382. doi:10.1007/s00018-013-1497-8. 24162934 PMC3961528

[20] Si A, Zhang S-W, Maleszka R. 2005. Effects of caffeine on olfactory and visual learning in the honey bee (Apis mellifera). Pharmacol Biochem Behav 82:664–672. doi:10.1016/j.pbb.2005.11.009. 16375953

[21] Muth F, Philbin CS, Jeffrey CS, Leonard AS. 2022. Discovery of octopamine and tyramine in nectar and their effects on bumblebee behavior. iScience 25:104765. doi:10.1016/j.isci.2022.104765. 35942103 PMC9356080

[22] Marchi IL, Palottini F, Farina WM. 2021. Combined secondary compounds naturally found in nectars enhance honeybee cognition and survival. J Exp Biol 224:jeb239616. doi:10.1242/jeb.239616. 33602677

[23] Prado SG, Collazo JA, Marand MH, Irwin RE. 2021. The influence of floral resources and microclimate on pollinator visitation in an agro-ecosystem. Agric Ecosyst Environ 307:107196. doi:10.1016/j.agee.2020.107196.

[24] Couvillon MJ, Al Toufailia H, Butterfield TM, Schrell F, Ratnieks FLW, Schürch R. 2015. Caffeinated forage tricks honeybees into increasing foraging and recruitment behaviors. Curr Biol 25:2815–2818. doi:10.1016/j.cub.2015.08.052. 26480843

[25] Peng T, Segers FHID, Nascimento F, Grüter C. 2019. Resource profitability, but not caffeine, affects individual and collective foraging in the stingless bee Plebeia droryana. J Exp Biol 222:jeb195503. doi:10.1242/jeb.195503. 31064857

[26] Koch H, Stevenson PC. 2017. Do linden trees kill bees? Reviewing the causes of bee deaths on silver linden (Tilia tomentosa). Biol Lett 13:20170484. doi:10.1098/rsbl.2017.0484. 28954857 PMC5627179

[27] Arathi HS, Bernklau E. 2021. Context-dependent effect of dietary phytochemicals on honey bees exposed to a pesticide, thiamethoxam. J Insect Sci 21:11. doi:10.1093/jisesa/ieab053. PMC8353980 34374762

[28] Balieira KVB, Mazzo M, Bizerra PFV, Guimarães Ar de JS, Nicodemo D, Mingatto FE. 2018. Imidacloprid-induced oxidative stress in honey bees and the antioxidant action of caffeine. Apidologie 49:562–572. doi:10.1007/s13592-018-0583-1.

[29] Richman SK, Maalouf IM, Smilanich AM, Marquez Sanchez D, Miller SZ, Leonard AS. 2022. A neonicotinoid pesticide alters how nectar chemistry affects bees. Funct Ecol 36:1063–1073. doi:10.1111/1365-2435.14016.

[30] Li Z, Huang Q, Zheng Y, Zhang Y, Li X, Zhong S, Zeng Z. 2022. Identification of the toxic compounds in camellia oleifera honey and pollen to honey bees (Apis mellifera). J Agric Food Chem 70:13176–13185. doi:10.1021/acs.jafc.2c04950. 36214176

[31] Bernklau E, Bjostad L, Hogeboom A, Carlisle A, H S A. 2019. Dietary phytochemicals, honey bee longevity and pathogen tolerance. Insects 10:14. doi:10.3390/insects10010014. 30626025 PMC6359238

[32] Mao W, Schuler MA, Berenbaum MR. 2013. Honey constituents up-regulate detoxification and immunity genes in the western honey bee Apis mellifera. Proc Natl Acad Sci USA 110:8842–8846. doi:10.1073/pnas.1303884110. 23630255 PMC3670375

[33] Palmer-Young EC, Tozkar CÖ, Schwarz RS, Chen Y, Irwin RE, Adler LS, Evans JD. 2017. Nectar and pollen phytochemicals stimulate honey bee (Hymenoptera: Apidae) immunity to viral infection. J Econ Entomol 110:1959–1972. doi:10.1093/jee/tox193. 28981688

[34] Tokarev YS, Huang W-F, Solter LF, Malysh JM, Becnel JJ, Vossbrinck CR. 2020. A formal redefinition of the genera Nosema and Vairimorpha (Microsporidia: Nosematidae) and reassignment of species based on molecular phylogenetics. J Invertebr Pathol 169:107279. doi:10.1016/j.jip.2019.107279. 31738888

[35] Strachecka A, Krauze M, Olszewski K, Borsuk G, Paleolog J, Merska M, Chobotow J, Bajda M, Grzywnowicz K. 2014. Unexpectedly strong effect of caffeine on the vitality of western honeybees (Apis mellifera). Biochemistry (Mosc) 79:1192–1201. doi:10.1134/S0006297914110066. 25540004

[36] Folly AJ, Koch H, Farrell IW, Stevenson PC, Brown MJF. 2021. Agri-environment scheme nectar chemistry can suppress the social epidemiology of parasites in an important pollinator. Proc Biol Sci 288:20210363. doi:10.1098/rspb.2021.0363. 34034519 PMC8150011

[37] Richardson LL, Adler LS, Leonard AS, Andicoechea J, Regan KH, Anthony WE, Manson JS, Irwin RE. 2015. Secondary metabolites in floral nectar reduce parasite infections in bumblebees. Proc Royal Soc B 282:20142471. doi:10.1098/rspb.2014.2471. PMC4345440 25694627

[38] Lu Y-H, Wu C-P, Tang C-K, Lin Y-H, Maaroufi HO, Chuang Y-C, Wu Y-L. 2020. Identification of immune regulatory genes in Apis mellifera through caffeine treatment. Insects 11:516. doi:10.3390/insects11080516. 32785078 PMC7469160

[39] Hsieh EM, Berenbaum MR, Dolezal AG. 2020. Ameliorative effects of phytochemical ingestion on viral infection in honey bees. Insects 11:698. doi:10.3390/insects11100698. 33066263 PMC7602108

[40] Raj CVS, Dhala S. 1965. Effect of naturally occurring xanthines on bacteria. Appl Microbiol 13:432–436. doi:10.1128/am.13.3.432-436.1965. 14325283 PMC1058267

[41] Al-Janabi AAHS. 2011. Potential activity of the purine compounds caffeine and aminophylline on bacteria. J Glob Infect Dis 3:133–137. doi:10.4103/0974-777X.81689. 21731299 PMC3125025

[42] Ceja-Navarro JA, Vega FE, Karaoz U, Hao Z, Jenkins S, Lim HC, Kosina P, Infante F, Northen TR, Brodie EL. 2015. Gut microbiota mediate caffeine detoxification in the primary insect pest of coffee. Nat Commun 6:7618. doi:10.1038/ncomms8618. 26173063 PMC4510693

[43] Geldert C, Abdo Z, Stewart JE, H S A. 2021. Dietary supplementation with phytochemicals improves diversity and abundance of honey bee gut microbiota. J Appl Microbiol 130:1705–1720. doi:10.1111/jam.14897. 33058297

[44] El Sanousi SM, El Sarag MSA, Mohamed SEY. 1987. 1987. Properties of Serratia marcescens isolated from diseased honeybee (Apis mellifera) larvae. Microbiology 133:215–219. doi:10.1099/00221287-133-1-215.

[45] Burritt NL, Foss NJ, Neeno-Eckwall EC, Church JO, Hilger AM, Hildebrand JA, Warshauer DM, Perna NT, Burritt JB. 2016. Sepsis and hemocyte loss in honey bees (Apis mellifera) infected with Serratia marcescens strain Sicaria. PLoS One 11:e0167752. doi:10.1371/journal.pone.0167752. 28002470 PMC5176276

[46] Raymann K, Coon KL, Shaffer Z, Salisbury S, Moran NA. 2018. Pathogenicity of Serratia marcescens strains in honey bees. mBio 9:e01649-18. doi:10.1128/mBio.01649-18. 30301854 PMC6178626

[47] Kwong WK, Moran NA. 2016. Gut microbial communities of social bees. Nat Rev Microbiol 14:374–384. doi:10.1038/nrmicro.2016.43. 27140688 PMC5648345

[48] Motta EVS, Powell JE, Leonard SP, Moran NA. 2022. Prospects for probiotics in social bees. Philos Trans R Soc Lond B Biol Sci 377:20210156. doi:10.1098/rstb.2021.0156. 35491599 PMC9058534

[49] Zheng H, Steele MI, Leonard SP, Motta EVS, Moran NA. 2018. Honey bees as models for gut microbiota research. Lab Anim (NY) 47:317–325. doi:10.1038/s41684-018-0173-x. 30353179 PMC6478020

[50] Schmidt TJ, Khalid SA, Romanha AJ, Alves TMA, Biavatti MW, Brun R, Da Costa FB, de Castro SL, Ferreira VF, de Lacerda MVG, Lago JHG, Leon LL, Lopes NP, das Neves Amorim RC, Niehues M, Ogungbe IV, Pohlit AM, Scotti MT, Setzer WN, Soeiro MNC, Steindel M, Tempone AG. 2012. The potential of secondary metabolites from plants as drugs or leads against protozoan neglected diseases, part I. Curr Med Chem 19:2128–2175. doi:10.2174/092986712800229023. 22414103

[51] Schmidt TJ, Khalid SA, Romanha AJ, Alves TMA, Biavatti MW, Brun R, Da Costa FB, de Castro SL, Ferreira VF, de Lacerda MVG, Lago JHG, Leon LL, Lopes NP, das Neves Amorim RC, Niehues M, Ogungbe IV, Pohlit AM, Scotti MT, Setzer WN, Soeiro MNC, Steindel M, Tempone AG. 2012. The potential of secondary metabolites from plants as drugs or leads against protozoan neglected diseases, part II. Curr Med Chem 19:2176–2228. doi:10.2174/092986712800229087. 22414104

[52] Abbott J. 2014. Self-medication in insects: current evidence and future perspectives. Ecol Entomol 39:273–280. doi:10.1111/een.12110.

[53] de Roode JC, Hunter MD. 2019. Self-medication in insects: when altered behaviors of infected insects are a defense instead of a parasite manipulation. Curr Opin Insect Sci 33:1–6. doi:10.1016/j.cois.2018.12.001. 31358187

[54] Simone-Finstrom M, Borba RS, Wilson M, Spivak M. 2017. Propolis counteracts some threats to honey bee health. Insects 8:46. doi:10.3390/insects8020046. 28468244 PMC5492060

[55] Raymann K, Shaffer Z, Moran NA. 2017. Antibiotic exposure perturbs the gut microbiota and elevates mortality in honeybees. PLoS Biol 15:e2001861. doi:10.1371/journal.pbio.2001861. 28291793 PMC5349420

[56] Evans JD, Schwarz RS. 2011. Bees brought to their knees: microbes affecting honey bee health. Trends Microbiol 19:614–620. doi:10.1016/j.tim.2011.09.003. 22032828

[57] Schwarz RS, Teixeira ÉW, Tauber JP, Birke JM, Martins MF, Fonseca I, Evans JD. 2014. Honey bee colonies act as reservoirs for two Spiroplasma facultative symbionts and incur complex, multiyear infection dynamics. Microbiologyopen 3:341–355. doi:10.1002/mbo3.172. 24771723 PMC4082708

[58] Budge GE, Shirley MDF, Jones B, Quill E, Tomkies V, Feil EJ, Brown MA, Haynes EG. 2014. Molecular epidemiology and population structure of the honey bee brood pathogen Melissococcus plutonius. ISME J 8:1588–1597. doi:10.1038/ismej.2014.20. 24599072 PMC4817608

[59] Genersch E. 2010. American foulbrood in honeybees and its causative agent, Paenibacillus larvae. J Invertebr Pathol 103:S10–S19. doi:10.1016/j.jip.2009.06.015. 19909971

[60] Alonso-Salces RM, Cugnata NM, Guaspari E, Pellegrini MC, Aubone I, De Piano FG, Antunez K, Fuselli SR. 2017. Natural strategies for the control of Paenibacillus larvae, the causative agent of American foulbrood in honey bees: a review. Apidologie 48:387–400. doi:10.1007/s13592-016-0483-1.

[61] Manson JS, Cook D, Gardner DR, Irwin RE. 2013. Dose-dependent effects of nectar alkaloids in a montane plant–pollinator community. J Ecol 101:1604–1612. doi:10.1111/1365-2745.12144.

[62] Mustard JA, Dews L, Brugato A, Dey K, Wright GA. 2012. Consumption of an acute dose of caffeine reduces acquisition but not memory in the honey bee. Behav Brain Res 232:217–224. doi:10.1016/j.bbr.2012.04.014. 22521838

[63] Singer MS, Mace KC, Bernays EA. 2009. Self-medication as adaptive plasticity: increased ingestion of plant toxins by parasitized caterpillars. PLoS One 4:e4796. doi:10.1371/journal.pone.0004796. 19274098 PMC2652102

[64] Karban R, English-Loeb G. 1997. Tachinid parasitoids affect host plant choice by caterpillars to increase caterpillar survival. Ecology 78:603–611. doi:10.1890/0012-9658(1997)078[0603:TPAHPC]2.0.CO;2.

[65] Emery O, Schmidt K, Engel P. 2017. Immune system stimulation by the gut symbiont Frischella perrara in the honey bee (Apis mellifera). Mol Ecol 26:2576–2590. doi:10.1111/mec.14058. 28207182

[66] Kwong WK, Mancenido AL, Moran NA. 2017. Immune system stimulation by the native gut microbiota of honey bees. R Soc Open Sci 4:170003. doi:10.1098/rsos.170003. 28386455 PMC5367273

[67] Wu Y, Zheng Y, Chen Y, Wang S, Chen Y, Hu F, Zheng H. 2020. Honey bee (Apis mellifera) gut microbiota promotes host endogenous detoxification capability via regulation of P450 gene expression in the digestive tract. Microb Biotechnol 13:1201–1212. doi:10.1111/1751-7915.13579. 32338446 PMC7264748

[68] Zheng H, Nishida A, Kwong WK, Koch H, Engel P, Steele MI, Moran NA. 2016. Metabolism of toxic sugars by strains of the bee gut symbiont Gilliamella apicola. mBio 7:e01326-16. doi:10.1128/mBio.01326-16. 27803186 PMC5090037

[69] Engel P, Martinson VG, Moran NA. 2012. Functional diversity within the simple gut microbiota of the honey bee. Proc Natl Acad Sci USA 109:11002–11007. doi:10.1073/pnas.1202970109. 22711827 PMC3390884

[70] Zheng H, Perreau J, Powell JE, Han B, Zhang Z, Kwong WK, Tringe SG, Moran NA. 2019. Division of labor in honey bee gut microbiota for plant polysaccharide digestion. Proc Natl Acad Sci USA 116:25909–25916. doi:10.1073/pnas.1916224116. 31776248 PMC6926048

[71] Koch H, Schmid-Hempel P. 2011. Socially transmitted gut microbiota protect bumble bees against an intestinal parasite. Proc Natl Acad Sci USA 108:19288–19292. doi:10.1073/pnas.1110474108. 22084077 PMC3228419

[72] Lang H, Duan H, Wang J, Zhang W, Guo J, Zhang X, Hu X, Zheng H. 2022. Specific strains of honeybee gut Lactobacillus stimulate host immune system to protect against pathogenic Hafnia alvei. Microbiol Spectr 10:e01896-21. doi:10.1128/spectrum.01896-21. 34985299 PMC8729767

[73] Steele MI, Motta EVS, Gattu T, Martinez D, Moran NA. 2021. The gut microbiota protects bees from invasion by a bacterial pathogen. Microbiol Spectr 9:e00394-21. doi:10.1128/Spectrum.00394-21. 34523998 PMC8557934

[74] Li JH, Evans JD, Li WF, Zhao YZ, DeGrandi-Hoffman G, Huang SK, Li ZG, Hamilton M, Chen YP. 2017. New evidence showing that the destruction of gut bacteria by antibiotic treatment could increase the honey bee’s vulnerability to Nosema infection. PLoS One 12:e0187505. doi:10.1371/journal.pone.0187505. 29125851 PMC5681286

[75] Motta EVS, Powell JE, Moran NA. 2022. Glyphosate induces immune dysregulation in honey bees. Anim Microbiome 4:16. doi:10.1186/s42523-022-00165-0. 35193702 PMC8862317

[76] Motta EVS, Mak M, De Jong TK, Powell JE, O'Donnell A, Suhr KJ, Riddington IM, Moran NA. 2020. Oral or topical exposure to glyphosate in herbicide formulation impacts the gut microbiota and survival rates of honey bees. Appl Environ Microbiol 86. doi:10.1128/AEM.01150-20. PMC7480383 32651208

[77] Motta EVS, Raymann K, Moran NA. 2018. Glyphosate perturbs the gut microbiota of honey bees. Proc Natl Acad Sci USA 115:10305–10310. doi:10.1073/pnas.1803880115. 30249635 PMC6187125

[78] Almeida AAP, Farah A, Silva DAM, Nunan EA, Glória MBA. 2006. Antibacterial activity of coffee extracts and selected coffee chemical compounds against Enterobacteria. J Agric Food Chem 54:8738–8743. doi:10.1021/jf0617317. 17090115

[79] Ibrahim S, Shukor MY, Syed MA, Rahman NAA, Khalil KA, Khalid A, Ahmad SA. 2014. Bacterial degradation of caffeine: a review. Asian J Plant Biol 2:19–28. doi:10.54987/ajpb.v2i1.84.

[80] Mazzafera P, Olsson O, Sandberg G. 1996. Degradation of caffeine and related methylxanthines by Serratia marcescens isolated from soil under coffee cultivation. Microb Ecol 31:199–207. doi:10.1007/BF00167865. 24185743

[81] Evans JD, Chen YP, Prisco G, Pettis J, Williams V. 2009. Bee cups: single-use cages for honey bee experiments. J Apic Res 48:300–302. doi:10.1080/00218839.2009.11101548.

[82] Kwong WK, Medina LA, Koch H, Sing K-W, Soh EJY, Ascher JS, Jaffé R, Moran NA. 2017. Dynamic microbiome evolution in social bees. Sci Adv 3:e1600513. doi:10.1126/sciadv.1600513. 28435856 PMC5371421

[83] Motta EVS, Moran NA. 2023. The effects of glyphosate, pure or in herbicide formulation, on bumble bees and their gut microbial communities. Sci Total Environ 872:162102. doi:10.1016/j.scitotenv.2023.162102. 36764553 PMC11050743

[84] Bolyen E, Rideout JR, Dillon MR, Bokulich NA, Abnet CC, Al-Ghalith GA, Alexander H, Alm EJ, Arumugam M, Asnicar F, Bai Y, Bisanz JE, Bittinger K, Brejnrod A, Brislawn CJ, Brown CT, Callahan BJ, Caraballo-Rodríguez AM, Chase J, Cope EK, Da Silva R, Diener C, Dorrestein PC, Douglas GM, Durall DM, Duvallet C, Edwardson CF, Ernst M, Estaki M, Fouquier J, Gauglitz JM, Gibbons SM, Gibson DL, Gonzalez A, Gorlick K, Guo J, Hillmann B, Holmes S, Holste H, Huttenhower C, Huttley GA, Janssen S, Jarmusch AK, Jiang L, Kaehler BD, Kang KB, Keefe CR, Keim P, Kelley ST, Knights D, et al. 2019. Reproducible, interactive, scalable and extensible microbiome data science using QIIME 2. 8. Nat Biotechnol 37:852–857. doi:10.1038/s41587-019-0209-9. 31341288 PMC7015180

[85] Martin M. 2011. Cutadapt removes adapter sequences from high-throughput sequencing reads. EMBnet J 17:10–12. doi:10.14806/ej.17.1.200.

[86] Amir A, McDonald D, Navas-Molina JA, Kopylova E, Morton JT, Zech Xu Z, Kightley EP, Thompson LR, Hyde ER, Gonzalez A, Knight R. 2017. Deblur rapidly resolves single-nucleotide community sequence patterns. mSystems 2:e00191-16. doi:10.1128/mSystems.00191-16. 28289731 PMC5340863

[87] Bokulich NA, Kaehler BD, Rideout JR, Dillon M, Bolyen E, Knight R, Huttley GA, Gregory Caporaso J. 2018. Optimizing taxonomic classification of marker-gene amplicon sequences with QIIME 2’s q2-feature-classifier plugin. Microbiome 6:90. doi:10.1186/s40168-018-0470-z. 29773078 PMC5956843

[88] Therneau TM. 2020. coxme: mixed effects Cox models, R package version 2.2-16. https://CRAN.R-project.org/package=coxme (2.2-16).

[89] Kassambara A, Kosinski M, Biecek P. 2020. survminer: drawing survival curves using “ggplot2,” R package version 0.4.7. https://CRAN.R-project.org/package=survminer (0.4.7).

[90] Fox J, Weisberg S. 2019. An R companion to applied regression, 3rd ed. Sage, Thousand Oaks, CA.

[91] Lenth R, Singmann H, Love J, Buerkner P, Herve M. 2019. emmeans: estimated marginal means, aka least-squares means (1.4.3.01). https://CRAN.R-project.org/package=emmeans.

[92] McMurdie PJ, Holmes S. 2013. phyloseq: an R package for reproducible interactive analysis and graphics of microbiome census data. PLoS One 8:e61217. doi:10.1371/journal.pone.0061217. 23630581 PMC3632530

[93] Oksanen J, Simpson GL, Blanchet FG, Kindt R, Legendre P, Minchin PR, O’Hara RB, Solymos P, Stevens MHH, Szoecs E, Wagner H, Barbour M, Bedward M, Bolker B, Borcard D, Carvalho G, Chirico M, Caceres MD, Durand S, Evangelista HBA, FitzJohn R, Friendly M, Furneaux B, Hannigan G, Hill MO, Lahti L, McGlinn D, Ouellette M-H, Cunha ER, Smith T, Stier A, Braak CJFT, Weedon J. 2019. vegan: community ecology package (2.5–6). https://CRAN.R-project.org/package=vegan.

[94] R Core Team. 2013. R: a language and environment for statistical computing (3.5.2). R Foundation for Statistical Computing, Vienna, Austria.

